# Meal Replacements for Weight Loss in Type 2 Diabetes in a Community Setting

**DOI:** 10.1155/2012/918571

**Published:** 2012-10-02

**Authors:** Jennifer B. Keogh, Peter M. Clifton

**Affiliations:** ^1^Division of Health Sciences, School of Pharmacy and Medical Sciences, University of South Australia, Adelaide, SA 5000, Australia; ^2^Nutritional Interventions, Baker IDI Heart and Diabetes Institute, North Terrace, Adelaide, SA 5000, Australia

## Abstract

*Background*. There is limited information on the effectiveness of meal replacements (MRs) as a weight-loss strategy in an unsupervised community setting. *Aim*. To evaluate the use of MR compared with a diet book for 6 months. *Subjects and Methods*. Obese subjects (*n* = 120) with type 2 diabetes mellitus were recruited from the community in Adelaide, South Australia, and randomised to intervention or control. Subjects in the intervention were advised to consume 2 MR/day for 3 months and 1 MR/day for 3 months and follow the manufacturers' instructions from printed material and the website. Subjects in the control arm were given a commercially available diet book. *Results*. Consumption of 2 MR for 3 months and 1 MR for the subsequent 3 months led to weight loss of 5.5 kg (5%) and a 0.26% decrease in HbA1c while the diet book group had a weight loss of 3 kg (3%) (*P* = 0.027 for difference between groups) and a decrease in HbA1c of 0.15% (between group ns) in those who completed the 6-month study. On intention-to-treat (last observation carried forward) weight loss at 6 months was 3.4 kg in MR and 1.8 kg in control (*P* = 0.07). Decreases in HbA1c were 0.22% and 0.12%, respectively (*P* = ns). HDL cholesterol increased by 4% in MR and decreased by 1% in control (*P* = 0.004). Blood pressure decreased equally in both groups. There were reductions in fasting glucose in both groups at 6 months with no changes in LDL-cholesterol or triglyceride concentrations. *Conclusion*. MR confers benefits in HbA1C reduction and weight loss at 6 months in those who completed the study.

## 1. Background

Cardiovascular disease and type 2 diabetes are strongly associated with obesity and there is very good evidence that weight loss lowers glucose, blood pressure, LDL cholesterol, and triglyceride [1]. The incidence of obesity in Australia and other developed countries is increasing with a subsequent swell of public interest in dietary strategies to reduce body weight [2]. Formulated meal replacements designed for weight loss represent a possible strategy for some individuals [3]. There is little evidence that this approach is effective when used without professional support nor is there evidence for how it compares to conventional dietary approaches used in the same context. In previous studies we have demonstrated that meal replacement provided excellent results that were not different to professional dietary advice at 3 and 6 months in overweight and obese subjects with an elevated triglyceride level [4]. We have also shown that high protein meal replacements can be very effective in long term in nondiabetic subjects and although we did not have a control group in this study the meal replacements would certainly be better than advice alone [3]. Meal replacements are being used in the Look Ahead Diabetes study in the USA as part of a comprehensive behavioural strategy and at 12 months a weight loss of 8.6% versus 0.8% in the control group had occurred [5] which had reduced to 4.7% and 1.1% at 4 years [6].

Other than the Wisconsin community study [7] most meal replacement trials have used either dietitian, physician, or nurse input in addition to information supplied with the meal replacements. However in practice most meal replacement products would be consumed without the assistance of dietitian input and feedback as they are freely available for purchase in community pharmacies and supermarkets. Our aims were to evaluate, under field conditions, a weight loss strategy of a meal replacement product compared with no treatment for 6 months. Our hypotheses were that the meal replacements would lead to a weight loss of 8–10 kg at 12 weeks which would be maintained at 6 months and would produce greater HbA1c lowering compared with standard dietary advice provided using a commercially available diet book. 

## 2. Subjects and Methods

One hundred and twenty overweight and obese subjects with type 2 diabetes mellitus were recruited from the Commonwealth Scientific and Industrial Research Organisation (CSIRO) database. Inclusion criteria were type 2 diabetes (HbA1c 6.5–12%), age 20–65 years, not greater than 140 kg (exceeded scale limit), with no abnormality of clinical significance on medical history and if female, not pregnant or breast feeding. Subjects had to understand the procedures involved and agree to participate in the study by giving full informed, written consent. All medication was allowable including insulin. Exclusion criteria were type 1 diabetes (by history only), history of heavy alcohol consumption (>5 STD drinks/day) and participant unable to cease alcohol consumption for study duration, widely fluctuating exercise patterns, frequent dining out (>2x/week) and unable to cease, inability to prepare meals or meet diet requirements, extended absences due to travel or other commitments or unable to comprehend or cope with study requirements. 

The following items if taken had to be kept stable during the study: prednisolone, cholesterol lowering agents, antihypertensive agents, and fish oil supplements.

### 2.1. Study Design

Following screening and selection from the CSIRO volunteer database, subjects were randomised into two groups: meal replacement (MR) or diet book control (DB) with 60 subjects in each group matched for age, sex, and BMI using the Clinstat randomisation program. The authors were blinded to treatment allocation until after the statistical analysis was performed. Subjects then commenced a 24-week active weight loss phase. The meal replacement group consumed 2 meal replacements (880 kilojoules each) and a low fat evening meal per day with at least 5 serves of fruit and vegetables/day (total approximately 5000 kJ). Meal replacements were provided at the CSIRO clinic at no charge every 4 weeks. All subjects were weighed every 4 weeks. Meal replacements Probiotec Formula WL (Probiotec Limited, Laverton North, VIC, Australia 3026) were used. The meal replacement group was given all the product material and access to the website. No dietary information was sought other than compliance to use of the meal replacement with a daily checklist.

**Figure 1:**
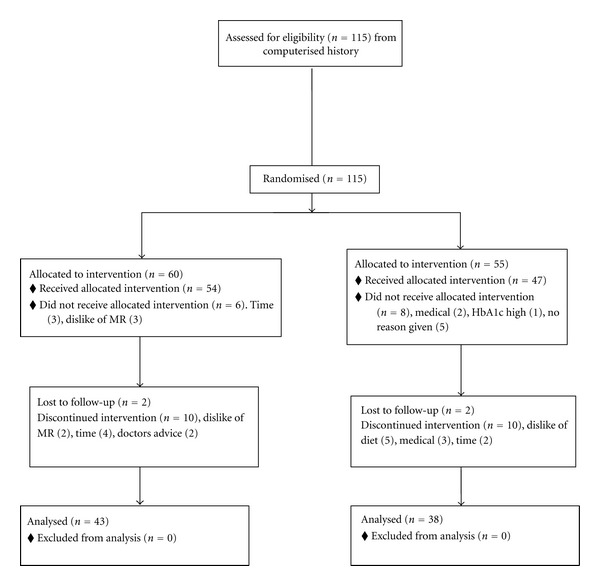
CONSORT flow diagram.

The control group were given the CSIRO Total Wellbeing Diet Book and were given no verbal advice. Vouchers to the value of the MRs were provided every 4 weeks. Control subjects were weighed at the same frequency as the meal replacement group. The study was approved by the CSIRO Food and Nutritional Sciences Human Ethics Committee and written, informed consent was obtained. A CONSORT statement is shown in Figure 1.

### 2.2. Measurements

Body weight was measured using an electronic digital scales (Mercury, AMZ14, Tokyo, Japan). Body composition (total fat mass (FM) and total fat free mass (FFM)) was measured using dual-energy X-ray absorptiometry (Lunar Prodigy; General Electric Corporation, Madison, WI, USA). Seated blood pressure was measured, after 5 minutes rest, using an automated sphygmomanometer (DINAMAP 8100, Criticon, Tampa, FL, USA).

### 2.3. Biochemistry

Plasma glucose, and serum lipids and insulin were measured using standard commercial assay kits as published previously [8]. HbA1c was measured using high-performance liquid chromatography at a certified commercial laboratory (Pathology SA, Adelaide, SA).

### 2.4. Statistical Analysis

Statistical analysis was performed using PASW for Windows (version 18.0, SPSS, Chicago, IL, USA). One-way analysis of variance (ANOVA) was used to compare between-group differences at baseline. The effects of time and group were assessed using repeated measures ANOVA with time as the within-subject factor and group as between-subject factor. A completers analysis was performed and there was also an intention to treat analysis for weight and HbA1c. Statistical significance was set at *P* ≤ 0.05. Data are presented as means ± standard deviation.

### 2.5. Power

We had enough power (80%, alpha 0.05) to detect a difference from control of 2 kg in weight, and a difference in LDL cholesterol of 0.2 mmol/L and a difference in HbA1c of 0.3%.

## 3. Results

### 3.1. Subjects

Forty three subjects (16 women, 27 men) MR subjects and 38 (15 women, 23 men) DB subjects completed 6-months. Baseline characteristics of those enrolled and the 6 months completers are shown in Table 1. Those who dropped out had a significantly greater BMI (36 kg/m^2^) than those who completed 6 months (33.7 kg/m^2^) (*P* = 0.021).

### 3.2. Weight

Weight of the subjects who completed 3 months of the study is shown in Table 2. Weight loss at 3 months was 5.5 kg versus 3.0 kg and at 6 months was 5.0 kg versus 3.0 kg in the 6-month completers group. This is very similar to the whole group at 3 months where the weight loss was 5.3 kg versus 2.7 kg (Table 3, *P* = 0.01, *n* = 87). The overall effect of both diets was significant (time *P* = 0.001) with a diet by time interaction of *P* = 0.016. The change in weight was significantly different between treatments at both 3 months (*P* = 0.001) and 6 months (*P* = 0.027). The change in weight was not predicted by age, sex, BMI, alcohol consumption, smoking, exercise pattern, nor postmenopausal status. Within the MR group the number of meal replacements consumed and initial BMI predicted the weight change at 6 months and accounted for 25% of the variance (*P* = 0.03). This also applied at 3 months and accounted for 32% of the variance (*P* = 0.002). Within the DB group gender was associated with weight loss with men losing more weight than women at 3 months (*P* = 0.029) but not at 6 months. 

On intention to treat analysis with last observation carried forward weight loss at 3 months was 4.0 ± 3.8 kg in the MR group versus 2.3 ± 2.3 kg in the DB group (*P* < 0.01) while at 6 months the weight loss was 3.4 ± 5.2 kg versus 1.8 ± 3.4 kg (*P* = 0.07).

### 3.3. Body Composition

Body composition is shown in Table 4. Bone mineral density BMD (*P* < 0.001) but not bone mineral content (total bone mass, bone mineral content (BMC)) changed with time with no differences between treatments. Fat mass and lean mass changed with time (*P* < 0.001) with a treatment interaction with fat mass (*P* = 0.04) such that fat mass fell more with MR (3.0 kg versus 1.3 kg). The change in fat mass was greater in MR in the legs (peripheral fat) (0.7 kg versus 0.0 kg at 6 months, *P* < 0.05) rather than the trunk (central fat) where the fat loss was not statistically different despite a difference of 2.1 kg versus 1.3 kg. In the android region the total fat (*P* = 0.051) and the fat/lean ratio was different between treatments falling 10% with MR and with no change in DB (*P* = 0.035). Smokers had enhanced the lean loss in the android region. There were no differences in the gynoid region.

On linear regression predictors of total fat change were postmenopausal status (negatively), HRT (positively), and exercise (positively), accounting for 16% of the variance in fat change (*P* = 0.013). For the 3-month fat change treatment also played a role along with HRT and postmenopausal status, accounting for 21% of the variance, *P* = 0.001. Lean mass loss at 6 months was directly related to starting weight and smoking (10% of variance, *P* = 0.015). At 3 months lean loss was higher in women and related to baseline lean mass. 

### 3.4. HbA1c

Blood tests for HbA1c at 6 months were obtained from 40 on MR and 34 on DB. Overall (using baseline, 3 months and 6 months) there was an effect of time (*P* = 0.035) and there was a diet by time interaction (*P* = 0.021) (Table 5). The change in HBA1c at 3 months was 0.49% for MR and 0% for DB (*P* = 0.015 for diet by time interaction) and at 6 months was 0.26% for MR and 0.15% for DB (*P* = 0.5 for diet by time interaction). There were reductions of drug dose in 6 volunteers in the MR group (5 of whom had falls in HbA1c at 6 months) and 2 reductions and 2 increases in the DB (which lead to further drops in HbA1c of 0.6% and 1% in these volunteers). Overall time (*P* < 0.001) treatment (*P* = 0.018) and dosage change (*P* = 0.015) were significant. Statistically dosage change had a very strong effect on HbA1c at 3 months which is when most of the changes occurred (treatment *P* = 0.001, dosage change *P* = 0.006, interaction *P* = 0.003) and a weaker effect at 6 months (treatment *P* = 0.06, dosage change *P* = 0.026, interaction *P* = 0.054). Those who had a dosage change at 6 months on MR had a fall in HbA1c of 1.2% (*n* = 6), which clearly would have been greater if the reduction had not occurred while those who had a dosage change on the diet book only had a change in HbA1c of 0.2% (*n* = 4). There was a reduction in the amount of insulin in 3 people on MR (1 stopped altogether) and there were 3 reductions in oral medication in the MR group. In the book group there was one reduction in insulin dosage and 1 reduction in medication dosage and 2 alterations in medication type.

For the change in HbA1c at 6 months both screening HbA1c and change in weight at 6 months together predicted 21% of the change in HbA1c (*P* = 0.001) while for the change in HbA1c at 3 months change in medication and treatment and screening HbA1c accounted for 25% of the variance (*P* = 0.001).

On intention to treat analysis with last observation carried forward fall in HbA1c at 3 months was 0.40 ± 0.83% in the MR group versus 0 ± 0.71% in DB (*P* < 0.01) while at 6 months the fall was 0.22 ± 0.74% versus 0.12 ± 0.66% (*P* = 0.5).

### 3.5. Lipids and Glucose

Cholesterol (*P* = 0.012), triglyceride (*P* = 0.0310), and glucose (*P* = 0.002) concentrations changed with both diets at 3 months and there were time by treatment interactions for HDL cholesterol only (*P* = 0.004) with rises in HDL for MR and fall in HDL for diet book. HDL cholesterol was significantly lower at all time points in the MR group (Table 6). At 6 months only glucose was lower than at baseline in both diets and there was a medication change (*P* = 0.014) interaction (Table 7). 

### 3.6. Blood Pressure

Both systolic blood pressure (SBP) and diastolic blood pressure (DBP) decreased significantly with time (SBP 129 ± 14 to 124 ± 13 to 128 ± 13 mmHg at 3 and 6 months, resp., *n* = 77, *P* < 0.001, DBP 76 ± 10 to 71 ± 9 to 73 ± 9 mmHg at 3 and 6 months, resp., *n* = 77, *P* < 0.001), but there was no effect of treatment type. 

## 4. Discussion

The main finding of the study, that set out to mirror a real world setting where individuals buy meal replacements from a supermarket or pharmacy, was that consumption of 2 meal replacements for 3 months and 1 meal replacement for a further 3 months led to greater weight loss of approximately 2 kg (5%) and a 0.11% decrease in HbA1c compared with control subjects who used a commercially available weight loss book. However on intention to treat analysis which reflects real world use of the meal replacements with many people discontinuing treatment by 6 months there was no difference seen in either weight or HbA1c compared with the weight loss book. If the meal replacements were purchased rather than provided, then drop out rates may have been even higher. There were no benefits on LDL cholesterol or triglyceride but HDL cholesterol was increased compared with the diet book group. Fasting triglyceride was not abnormal to begin with and the 5% weight loss was too low to alter it significantly. Fasting glucose was decreased to a small degree in both groups reflecting reduced food intake. The overall weight loss in the meal replacement group of 5.5 kg in those who completed the study is clinically significant as is the fall in HbA1c of 0.3% from baseline and the changes are comparable to those seen in other studies [9–14].

Meal replacements are used as a weight loss strategy in people with and without type 2 diabetes and have become a strategy of choice when substantial weight loss is needed [9–11]. In a systematic review of controlled trials of lifestyle interventions Brown et al. observed that when meal replacements were added to a low-fat diet there was a significant improvement in weight loss of 5.4 kg [10]. In people with diabetes meal replacements are most often used as part of a multidisciplinary weight management program as they are a viable and potentially cost-effective solution to weight management in type 2 diabetes [9]. 

Intentional weight loss in people with diabetes is very effective at reducing cardiovascular risk. In a systematic review Aucott et al. (2004) found that people with diabetes who lost weight intentionally significantly reduced their mortality risks by 25% with weight loss of 9–13 kg being most protective [12]. Therefore development and use of effective weight loss strategies are very important in people with diabetes.

A number of studies have examined the use of meal replacements in this context [13–15].

In a study of 57 subjects with type 2 diabetes comparing meal replacements with an exchange diet plan for 12 weeks Yip et al. (2001) [13] found that weight loss in the two meal replacement groups were greater (6.4% and 6.7%, resp.) compared with weight loss in the diet plan group (4.9%) although this was not significant. In addition, fasting glucose, total cholesterol, and low-density lipoprotein cholesterol levels were reduced in the meal replacement groups and the glucose change was significant compared with the diet plan group (*P* = 0.01), but HbA1c was not different (0.2% difference between groups). Similarly Li et al. (2005) [14] in a longer 12-month study found that percentage weight loss was greater in the meal replacement group (4.6%) than in diet plan group (2.3%) although BMI change was not significant (*P* = 0.07). Fasting glucose was reduced in meal replacements group at 6 months and HbA1c level improved in MR at 6 months compared to the diet plan group by 0.49% (*P* = 0.1 for comparison), but this difference had disappeared by 12 months (0.15%, *P* = 0.6) and neither group had a significant change in HbA1c compared to baseline. Each participant in both groups received individual consultation with a registered dietitian at baseline, weeks 2, 4, 6, 8 and then monthly for the duration of the 1-year study and 77 out of 104 subjects completed the study. More subjects in meal replacements group reduced their use of medications. In a study of 119 subjects Cheskin et al. (2008) found that weight loss on a meal replacement program for 34 weeks with 1-year followup was greater than on a standard diet with more subjects 40% versus 12% achieving ≥5% weight loss [16]. Dropout was very large with only 48 completing 34 weeks and 33 completing 86 weeks. HbA1c dropped by 0.28% in the MR group versus an increase of 0.32% in the standard diet (*P* = 0.06). These HbA1c results are very similar to ours as were the changes in medication with seven more individuals reducing drugs in the MR group than in the SD group. Dosage increases were similar in both groups. In a report by Ditschuneit (2006) patients with diabetes on a meal replacement program achieved weight loss of 5.2% and 4.4% of their body weight at 6 and 12 months, respectively, [15]. In comparison patients on a diet plan achieved weight loss of 2.9% and 2.4% at 6 and 12 months. All these studies involved a dietitian in the program delivery.

It would appear that the immediate reduction in carbohydrate using the protein-rich meal replacements was the most important cause of a fall in HbA1c at 3 months in the MR group while the 2 kg weight loss in the DB group was not large enough or had not been present for long enough to change HbA1c. The regain of some of the lost weight as well as the reduction to one MR sachet per day may be the reason for the rise in HbA1c in the MR group at 6 months and lack of difference between the 2 groups at this time point. Medication dosage reduction clearly had an effect on the results as well, particularly in the MR group. The changes in HbA1c are very comparable with the published literature. Greater weight loss is required to maintain the early changes in HbA1c.

In conclusion use of meal replacements for 6 months can increase weight loss by 2.5 kg compared with nonprofessional advice from a diet book, but HbA1c was not significantly different between the groups. In order to achieve a greater reduction in HbA1c professional advice is probably required, although this is not a guarantee of success as the Li study showed no significant effect on HbA1c at 12 months in either group despite extensive professional advice.

## Figures and Tables

**Table 1:** Baseline characteristics of subjects enrolled and subjects who completed 6 months.

Baseline characteristics	All randomized *n* = 115	6-Month completers *N* = 81
Age (yr)	60.9	61.7
Women/men	49/66	30/50
Height (m)	1.71	1.71
Weight (kg)	100.8	100.7
BMI (kg/m^2^)	34.4	33.7*
HbA1c (%)	6.9	6.8
Alcohol consumers (%)	53	54
Smokers (%)	7	6
Exercise (%)	70	74
No diabetes medication (%)	25	17
Insulin (%)	14	8
1 drug (%)	37	22
2 drugs (%)	41	27
3 drugs (%)	9	5
4 drugs (%)	1	1
Antihypertensive drugs (%)	82	55
Cholesterol-lowering drugs (%)	75	52

Data are mean ± SD.

*P < 0.05.

**Table 2:** Subjects who completed 3 months.

Weight (kg)	Baseline		3 months	
Treatment	MR	DB	MR	DB
Mean ± SD	103.3 ± 16.6	99.7 ± 16.5	98.0 ± 16.5^*≠*^	96.5 ± 17.0

Data are mean ± SD.

MR: *n* = 47, DB: *n* = 40.

^*≠*^ *P* < 0.01 time by diet interaction.

**Table 3:** Subjects who completed 6 months.

Weight (kg)	Baseline		3 months		6 months	
Treatment	MR	DB	MR	DB	MR	DB
mean ± SD	102.4 ± 15.4	98.8 ± 16.7	96.9 ± 15.3^*≠*^	95.7 ± 16.9	97.45 ± 15.94*	95.8 ± 17.1

Data are mean ± SD.

MR: *n* = 43, DB: *n* = 37.

^*≠*^ *P* < 0.01 time by diet interaction.

^∗^ *P* < 0.05 time by diet interaction.

**Table 4:** Bone density, bone mineral content, fat and lean mass, by dual X-ray absorptiometry at baseline 3 and 6 months in subjects who completed the study.

	Baseline		3 months		6 months	
	MR	DB	MR	DB	MR	DB
BMD	1.34 ± 0.10	1.34 ± 0.12	1.32 ± 0.10	1.31 ± 0.12	1.34 ± 0.10	1.34 ± 0.12
BMC	3.38 ± 0.58	3.34 ± 0.66	3.37 ± 0.56	3.35 ± 0.65	3.37 ± 0.55	3.34 ± 0.67
Fat mass (kg)	38.9 ± 8.5	35.6 ± 9.8	35.5 ± 8.6*	33.8 ± 9.7	35.9 ± 9.2*	34.3 ± 9.9
Lean mass (kg)	58.6 ± 11.9	57.1 ± 9.6	57.2 ± 11.8	55.8 ± 9.8	57.3 ± 11.8	55.9 ± 8.9

Data are mean ± SD.

MR: *n* = 40, DB: *n* = 34, *P < 0.01 time by diet interaction.

**Table 5:** HbA1c.

Baseline		3 months		6 months	
MR	DB	MR	DB	MR	DB
7.3 ± 1.0%	7.1 ± 1.0%	6.8 ± 0.8%*	7.0 ± 1.1%	7.0 ± 1.0%	6.9 ± 0.9%

Data are mean ± SD.

MR: *n* = 40, DB: *n* = 34.

^∗^ *P* < 0.05 time by diet interaction at 3 months.

**Table 6:** Lipids and glucose at baseline and 3 months.

	Baseline		3 months	
	MR	DB	MR	DB
Total cholesterol (mmol/L)*	4.12 ± 1.12	4.22 ± 0.86	3.98 ± 1.06	4.06 ± 0.86
Triglycerides*	1.56 ± 0.50	1.57 ± 0.81	1.36 ± 0.58	1.48 ± 0.64
HDL cholesterol (mmol/L)	1.05 ± 0.23	1.20 ± 0.30	1.09 ± 0.29	1.15 ± 0.29
LDL cholesterol (mmol/L)*	2.36 ± 0.99	2.34 ± 0.80	2.28 ± 0.97	2.23 ± 0.77
Glucose (mmol/L)*	7.80 ± 1.63	7.65 ± 1.46	7.24 ± 1.49	7.33 ± 1.73

Data are mean ± SD.

MR: *n* = 47, DB: *n* = 40.

^∗^ *P* < 0.05 main effect of time.

**Table 7:** Lipids and glucose at baseline and 6 months.

	Baseline		6 months	
	MR	DB	MR	DB
Total cholesterol (mmol/L)	4.10 ± 1.15	4.20 ± 0.87	4.31 ± 1.15	4.18 ± 0.89
Triglyceride (mmol/L)	1.56 ± 0.54	1.57 ± 0.84	1.66 ± 0.75	1.58 ± 0.79
HDL cholesterol (mmol/L)	1.05 ± 0.22	1.22 ± 0.30	1.09 ± 0.26	1.21 ± 0.31
LDL cholesterol (mmol/L)	2.34 ± 1.01	2.30 ± 0.82	2.46 ± 0.99	2.25 ± 0.72
Glucose (mmol/L)*	7.85 ± 1.70	7.66 ± 1.52	7.48 ± 2.02	7.08 ± 1.30

Data are mean ± SD.

MR: *n* = 40, DB: *n* = 35.

^∗^ *P* < 0.05 main effect of time.
